# Preclinical Development of Seriniquinones as Selective Dermcidin Modulators for the Treatment of Melanoma

**DOI:** 10.3390/md20050301

**Published:** 2022-04-28

**Authors:** Amanda S. Hirata, James J. La Clair, Paula C. Jimenez, Leticia Veras Costa-Lotufo, William Fenical

**Affiliations:** 1 Department of Pharmacology, Institute of Biomedical Sciences, University of São Paulo, São Paulo 05508-900, Brazil; amanda.hirata@usp.br; 2 Department of Chemistry and Biochemistry, University of California, San Diego, CA 92093-0358, USA; 3 Institute of Marine Science, Federal University of São Paulo, Santos 11070-100, Brazil; pcjimenez@unifesp.br; 4 Center for Marine Biotechnology and Biomedicine, Scripps Institution of Oceanography, University of California, San Diego, CA 92093-0204, USA

**Keywords:** marine natural products, melanoma, seriniquinone, dermcidin, autophagy, apoptosis

## Abstract

The bioactive natural product seriniquinone was discovered as a potential melanoma drug, which was produced by the as-yet-undescribed marine bacterium of the rare genus *Serinicoccus*. As part of a long-term research program aimed at the discovery of new agents for the treatment of cancer, seriniquinone revealed remarkable in vitro activity against a diversity of cancer cell lines in the US National Cancer Institute 60-cell line screening. Target deconvolution studies defined the seriniquinones as a new class of melanoma-selective agents that act in part by targeting dermcidin (DCD). The targeted DCD peptide has been recently examined and defined as a “pro-survival peptide” in cancer cells. While DCD was first isolated from human skin and thought to be only an antimicrobial peptide, currently DCD has been also identified as a peptide associated with the survival of cancer cells, through what is believed to be a disulfide-based conjugation with proteins that would normally induce apoptosis. However, the significantly enhanced potency of seriniquinone was of particular interest against the melanoma cell lines assessed in the NCI 60-cell line panel. This observed selectivity provided a driving force that resulted in a multidimensional program for the discovery of a usable drug with a new anticancer target and, therefore, a novel mode of action. Here, we provided an overview of the discovery and development efforts to date.

## 1. The Discovery of Seriniquinone and its Early Bioactivity

**Figure 1:**
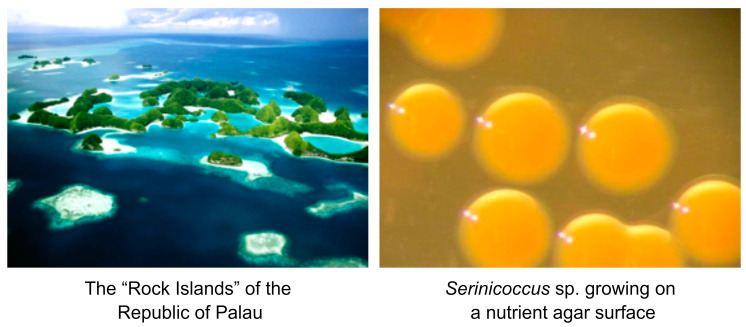
Aerial view of the Rock Islands of Palau where *Serinicoccus* was discovered (**left**) and the Gram-positive bacterium *Serinicoccus* sp. growing on a seawater-based nutrient agar surface (**right**).

With support from the US National Cancer Institute, an expedition of the Fenical group to the islands of Palau in the South Pacific was undertaken. Here, the goal was to isolate the unique marine bacteria which was found in both shallow and deep-water sediments. One sediment sample, when cultivated in a seawater-based nutrient medium yielded a unique Gram-positive bacterium, which is identified by 16S rDNA sequence methods as a member of the rare marine genus *Serinicoccus* (Figure 1). As part of this project, we cultured this bacterium and evaluated the whole culture organic extract against HCT-116 colon carcinoma using our in-house in vitro assay. The activity of the organic extract against HCT-116 was measured as LD_50_ value of 0.4 μg/mL, a modest but significant level of cytotoxicity.

**Figure 2:**
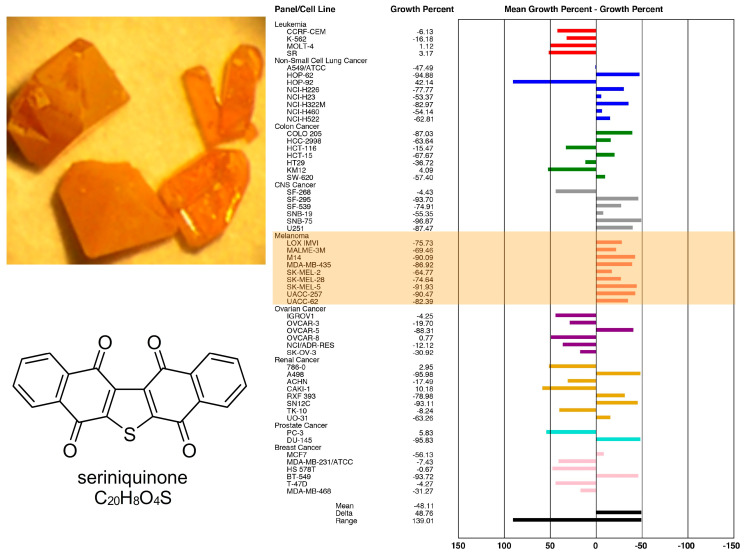
Crystals of seriniquinone as isolated and the chemical structure assigned (**left**), as well as activity data on seriniquinone (**right**). Bioassay results for seriniquinone against the 60-cell line panel offered by the Developmental Therapeutics Program at the NCI. Melanoma cell lines are highlighted in orange. A few other cell lines were also more sensitive to seriniquinone.

Members of the genus *Serinicoccus* have never been chemically explored. Therefore, we were intrigued when we found relevant cytotoxic activity from the crude extract. Cytotoxicity-guided purification led to the isolation of highly aromatic metabolite with the unusual molecular formula C_20_H_8_O_4_S. With limited structures that fit this formula, we were readily able to assign the structure of seriniquinone (Figure 2).

While the extract revealed only moderate cytotoxicity, when the pure compound was subjected to the NCI 60-cell line evaluation, we were excited to see a significant degree of selectivity (100–1000× enhanced potency) toward most cell lines of melanoma cancers (Figure 2).

On this basis, NCI requested the compound for their hollow fiber in vivo bioassay. Unfortunately, seriniquinone is highly insoluble that it could not be formulated to achieve an injectable dose. As a result, we realized that derivatives of seriniquinone were needed to improve on its aqueous solubility. Nonetheless, given the in vitro selectivity of this agent, we continued to explore its chemistry and cancer bioactivity. This started by developing synthetic methods to prepare seriniquinone and develop analogues of it, with the goal of identifying a lead compound with improved solubility and biological activity.

**Figure 3:**
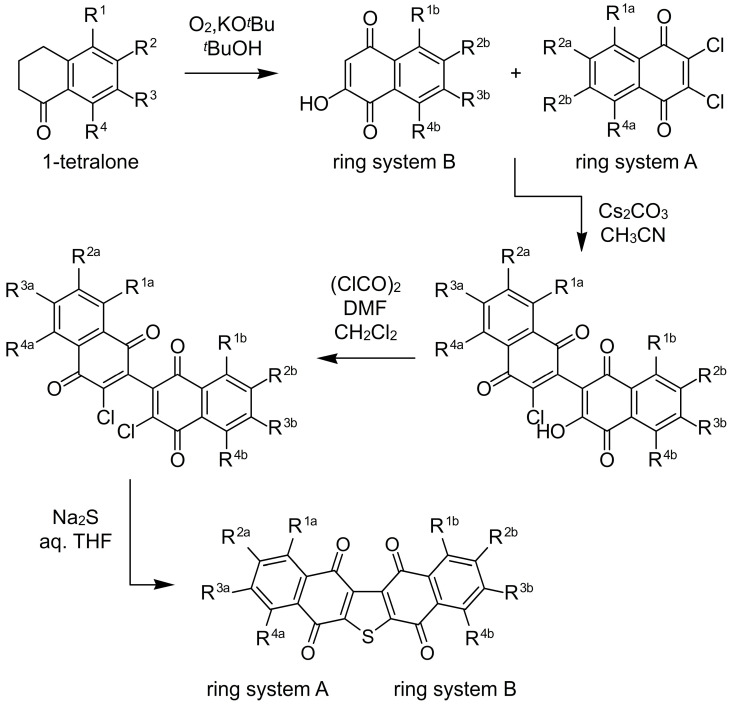
Synthetic approach to the seriniquinones. To date, a central synthetic approach has been established that enabled analogue preparation at four positions within each of the two ring systems. Seriniquinone (shown in Figure 2) is represented by R^1a−4a^ = H and R1^b−4b^ = H.

## 2. Facile Synthetic Access Enables Detailed Structure–Activity Relationship (SAR) Studies

One of the most daunting tasks with many natural product leads arises from the inability to obtain sufficient supplies of these materials to complete a detailed evaluation of their bioactivities. This process is typically evaluated through a series of studies commonly referred to as structure–activity relationship (SAR) analyses. To accomplish this step, one needs to develop a facile route for chemical synthesis and subsequent derivatization. Fortunately, our team was able to develop a concise route to prepare a wide array of analogues. As shown in Figure 3, we were able to prepare derivatives at multiple positions on the two-aryl rings in seriniquinone. The approach started by oxidation of the corresponding 1-tetralone to prepare the ring system B. Then, this was coupled to ring system A, a 2,3-dichlorinated-1,4-napthylquinone, by a basic condensation, optimally conducted with cesium carbonate in anhydrous CH_3_CN in the dark [1]. Thereafter, the resulting product was converted to its corresponding dichloride, and then treated with Na_2_S in aqueous THF to afford a seriniquinone analogue (Figure 3).

**Figure 4:**
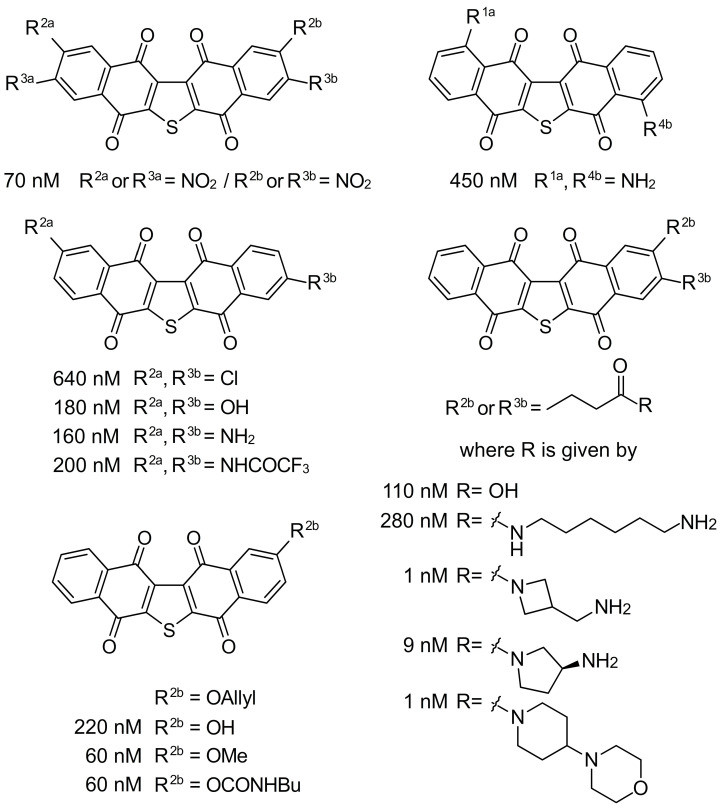
Exemplary analogues of seriniquinone prepared to date. IC_50_ values are provided for each compound against Malme-3M cells. Under these conditions, the parent seriniquinone (R^1a^–R^4a^ = H and R^1b^–R^4b^ = H) displayed an IC_50_ value of 60 nM.

This four-step procedure has been subsequently used to prepare a number of mono- and di-functionalized ring A and ring B analogues. Our first series of analogues were prepared through Friedel–Crafts reactions on seriniquinone. Seven derivatives were prepared and evaluated [1], that allowed us to explore functionalization at R^1a^–R^4a^ in ring A and R^1b^-R^4b^ in ring B. Unfortunately, this approach led to complex mixtures of isomers that were only obtained as pure compounds by complex chromatographic purification. However, from these mixtures, we were able to obtain two sets of single isomeric materials with dual substitutions at R^1a^ and R^4a^ and R^2a^ and R^3b^. Similar to the parent seriniquinone (R^1a^–R^4a^ = H and R^1b^–R^4b^ = H), these materials were highly insoluble in organic or aqueous media, complicating their usage. To solve this problem, we targeted analogues that contained larger alkyl functions within the aryl groups.

**Figure 5:**
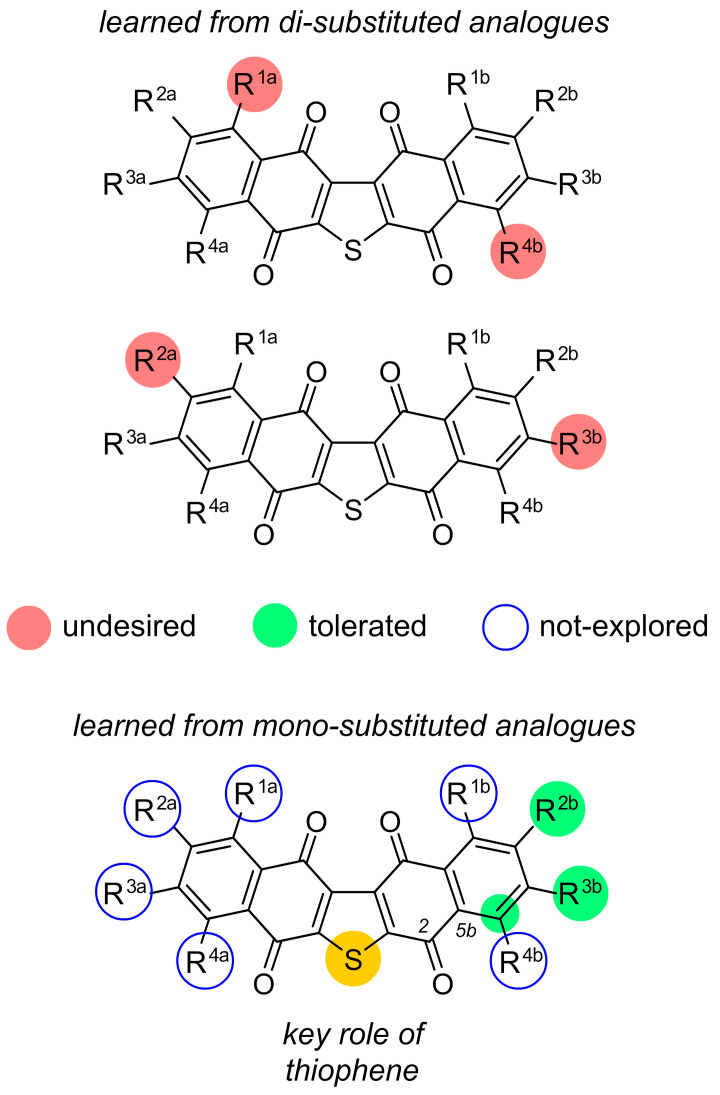
Current structure–activity relationship (SAR) maps obtained from mono- and di-substituted analogues. Undesired (red) depict modifications whose activity was significantly less than seriniquinone (R^1a^–R^4a^ = H and R^1b^–R^4b^ = H) (<50 nM in Malme-3M cells). Tolerated (green) denote analogues whose activity was similar to that of seriniquinone (R^1a^–R^4a^ = H and R^1b^–R^4b^ = H) (i.e., 60 ± 10 nM in Malme-3M cells). Undesired modifications that led to a reduction in activity are shown in red circles (≥100 nM in Malme-3M cells). An unfilled blue circle depicts unexplored sites.

In 2014, we reported the synthesis of a class bearing a carboxylic acid tail at R^2b^ and R^3b^ [2]. Unfortunately, the developed synthesis provided a mixture of the R^2b^ and R^3b^ isomers. From this scaffold, we were able to prepare multiple amide conjugates, which enabled a detailed evaluation at these positions [2]. From this set of analogues, three compounds were found with single digit nM activity (Figure 4), one of which contained the 4-(piperidin-4-yl) morpholine group that assisted with solubilization. Soon thereafter [1], we developed a route to prepare single isomers bearing a phenolic group at R^2b^. Here, we were able to prepare *O*-alkyl (allyl and methyl) and *N*-butylcarbamate derivatives, which displayed activities comparable to the parent seriniquinone with IC_50_ values at 60 nM. While far from complete, the data have started to map the modifications that SAR tolerated within this class. As shown in Figure 5, we have learned that analogues bearing dual substitutions with electron donating groups (OH or NH_2_) or halogens (Cl) do not present viable activity. Further data have been obtained by gaining access to *mono*-substituted analogues. To date, we have been able to demonstrate that both alkyl and alkoxy substitutions are viable when installed at positions R^2b^ and R^3b^. We have found that mono-substituted analogues not only provide viable activity, but also improve solubility. Breaking the symmetry of the seriniquinone structure (addition of a functional group at R^1a^–R^4a^ or R^1b^–R^4b^ disrupts the π–π stacking possible in the crystalline state of seriniquinone, where R^1a^–R^4a^ = H or R^1b^–R^4b^ = H) not only provides a means to enhance solubility, but also provides analogues with improved activities. To date, these studies have identified at least two analogues with activities ≥9 nM against Malme-3M. Recently, a team led by Fukuda described a biological transformation approach to expand the set of analogues [3]. Here, their team demonstrated that ring-opened analogs arising by Baeyer–Villiger, which is similar to the oxidation of C2 carbonyl and aryl group at C6B (see the labeled structure at the bottom of Figure 5) displayed viable activity against melanoma cell lines. These ring-opened analogues suggest an entirely new class of dihydronaphthothiophenes and further expand the utility of seriniquinones.

## 3. A Unique Mode of Action: Dermcidin

As seriniquinone was growing in interest for its melanoma selectivity, we invested in defining its mode of action (MOA). Seriniquinone, with inherent fluorescence, was incubated with HCT-116 colon carcinoma cells. In addition, changes in the incorporation during the cytotoxicity time course were monitored by confocal microscope [2]. Within 1 h, seriniquinone localized within the endoplasmic reticulum (ER). Then at 6 h, the compound transitioned into forming autophagosomes as the cells started to enter autophagocytosis (further confirmed by conversion of LC3A-I to LC3A-II and LC3B-I to LC3B-II) [2]. Counterstaining was used to validate these subcellular localization events. Next, we examined the effects of seriniquinone on the cell cycle. FACS analyses indicated an increasing number of cells with a subdiploid DNA, suggesting a concentration dependent DNA fragmentation. The remaining cells were blocked at the S to G2 phase, suggesting that the treated cells were dying and not re-entering the cell cycle.

Seriniquinone failed to induce DNA fragmentation using conventional in vitro assays. Therefore, we turned to apoptotic markers for further exploration. Treatment of HCT-116 cells with seriniquinone induced the cleavage of caspases-3, -7, and -9, and of PARP, suggesting that seriniquinone triggers apoptosis through a caspase-9 dependent event, leading to DNA fragmentation [2]. Given these interesting results, we next turned to screen for potential biological targets of seriniquinone. Hoping to apply the immunofluorescent (IAF) probe [4] precipitation method [5], we realized that we needed a suitable fluorescent tag precursor. Therefore, we defined a synthetic plan to disrupt the symmetry of the seriniquinone molecule by installing a readily functionalizable side-chain.

Treatment of HCT-116 cells with the fluorescently labeled seriniquinone [2] resulted in the same intracellular localization, which is followed by induction of autophagy and apoptosis as seen by the parent compound (both location and rate). Over multiple repetitions of immunoprecipitation, we obtained a series of proteins ranging from 30–250 kDa in a dose dependent manner when incubating with the IAF-labeled probe at 5–50 µM concentrations. Using trypsin digestion coupled with LC–MS/MS analyses to identify the proteins in the more abundant bands, we found that a protein band at 50 kDa contained peptides corresponding to glyceraldehyde 3-phosphate dehydrogenase (GADPH) along with a second set of peptides corresponding to the 30 aa PIF core, 13 aa propeptide, and 47 aa DCD-1 peptide regions of dermcidin (DCD). Then, the presence of these proteins was confirmed by Western blotting. Similarly, each of the examined bands contained the peptide fragments, which are derived from a given protein, such as actin or HSP70, along with fragments from dermcidin. In these studies, DCD was linked to these proteins by means of a disulfide bond, which was further released upon treatment with iodoacetamide. Treatment of the cell lysates with iodoacetamide before immunoprecipitation resulted in the isolation of a low molecular weight band corresponding to DCD. This observation indicated that the immunoprecipitated bands were the result of a disulfide-linked DCD protein. In these experiments, we observed the isolation of DCD, thereby suggesting that DCD was indeed a primary target of the IAF-labeled seriniquinone probe and, therefore, a putative target of the natural compound, as well.

With these data at hand, we examined the reactivity between seriniquinone and DCD in vitro. Using the commercially available 47 amino acid DCD-1 as a model, we found that exposure to seriniquinone as well as other analogues in Figure 4 covalently modified DCD-1. These studies provided strong support for the formation of the unique trimeric covalent-adduct between seriniquinone, DCD, and modulated-proteins, such as actin, GADPH, and HSP70.

## 4. Complex Pharmacology

**Figure 6:**
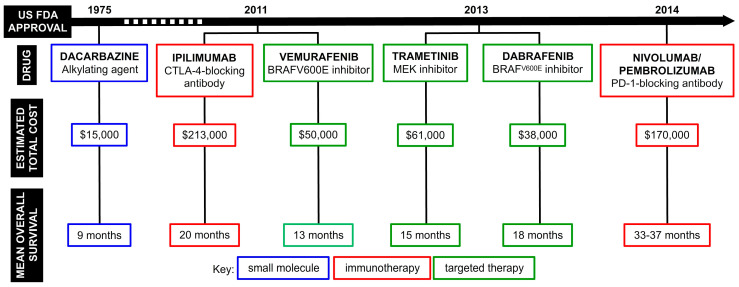
Timeline of melanoma therapy showing the mainly used drugs, along with the mean overall survival archived by patients and the total cost of the treatment per patient [16,17,18,19,20,21,22,23].

As previously mentioned, seriniquinone presented selectivity for melanoma cells and this dangerous type of cancer that arises from the skin needs urgent attention. The incidence of melanoma rises every year and, according to the Global Cancer Observatory, there were 324,635 new cases reported and 57,043 deaths in 2020 alone [6]. When the tumor is localized, surgical removal is the primary intervention. However, adjuvant chemotherapy is necessary once it metastasizes [7]. Dacarbazine, an alkylating agent, was the first and only drug approved for melanoma treatment. In addition, it has been the standard of care for over 30 years [8], although less than 30% of patients respond [9,10]. After all these orphan years, new therapeutic options with less toxicity and higher efficacy, such as immune and targeted therapies, emerged as promising solutions. Monotherapies of various cytokines (IFN-α and IL-2) and antibodies (ipilimumab, nivolumab, and pembrolizumab) improved the overall survival, but presented limited response rates and severe side effects [11,12,13]. The discovery of the high frequency of mutation in melanoma BRAF protein (over 50% of cases show BRAF^V600E^) set off the development of target-specific drugs, a strategy that was expected to overcome all barriers of toxicity and resistance faced until then [14]. Despite the fast tumor regression with vemurafenib and dabrafenib, due to their specificity to block the mutated BRAF protein that constitutively activates the MAPK driven proliferation, drug resistance appears after a short period of time for the majority of patients [15]. Given this scenario, several combined therapeutic regimens have been explored, but there is still the need for more efficient and cheaper pharmacological alternatives. Figure 6 depicts the timeline, total costs, and overall survival summary for the main monotherapy options that are currently available for melanoma.

Once the selective cytotoxicity of seriniquinone toward melanoma cell lines was observed, it was found that the compound was bound to a novel molecular target, one that was never pharmacologically explored before. Consequently, this compound emerged as both a potential treatment for melanoma and a tool to help in elucidating the role of dermcidin in this type of cancer. The cytotoxic autophagy induced by seriniquinone is a singular phenomenon first observed by Trzoss et al. [2] in colon carcinoma cell line (HCT-116) and in a BRAF mutant melanoma cell line (Malme-3M). Several proteins that participate in autophagy and apoptosis pathways can interact and trigger or block these phenomena [24], but how seriniquinone leads to either mechanism after the interaction with dermcidin is not completely understood. As a matter of fact, the value of autophagy as an anticancer strategy is the subject of an age-old debate. Under physiological conditions, this process can be activated by unfavorable conditions, such as nutrient depletion, enabling cells to function and survive with merely a basal energetic supply until they return to homeostasis. Similarly, in the context of cancer, autophagy can send cells into survival mode, allowing the cells to endure certain levels of stress and, therefore, favor cancer progression [25,26]. On the other hand, there are reports that show cytotoxic compounds which trigger autophagy may further lead to cell death [27,28,29], by apoptosis or otherwise. Further details regarding the complex interaction between the autophagy and apoptosis machineries are unknown, but we do know that the crosstalk exists since they share common components, regulators, and pathways that lead to the cell death [30]. In addition, this new mechanism of action may contribute to overcoming melanoma resistance.

Hirata et al. [31] expanded the studies on seriniquinone effects for other melanoma cell lines, including BRAF mutants (SK-MEL-28, SK-MEL-19, WM293A, MM200, 501MEL) and a NRAS mutant melanoma cell line (SK-MEL-147). NRAS mutation is the second most common among melanoma patients, yielding a more aggressive phenotype than BRAF mutants [32]. This is an interesting characteristic since it predicts that seriniquinone can act independently of the mutation, which distinguishes it from most of the currently available drugs. In addition to inducing caspase-mediated apoptosis, it was observed that the concomitant treatment of seriniquinone and an autophagy inhibitor altered cell responses. For the BRAF mutant, this blockage sensitized the cells to seriniquinone treatment, as expected and already explored for other cytotoxic compounds [33,34,35]. This effect improvement may be related to the high basal demand for autophagocytosis to maintain the proper mitochondrial function in BRAF^V600E^ cells [36,37]. However, the NRAS^Q61R^ melanoma, for which the basal autophagy was found to be suppressed, led to a serinoquinone-resistant phenotype under autophagy inhibition. In the literature, there are only a few studies describing similar phenomena [38,39]. Therefore, further studies are necessary to better elucidate this effect.

As suggested by the compound’s name, seriniquinone is classified as a napthoquinone, a chemical structure found in many other natural products. In general, this class of organic compounds has classical biological properties of DNA binding and ROS formation, as observed with the approved anticancer agent doxorubicin [40,41]. Hammons et al. [1] demonstrated that seriniquinone is an exception to this rule; it did not interact with DNA nor did it lead to ROS generation, for reasons still unknown. In addition to all of the uncommon mechanisms and effects elicited by this compound, these features only reinforce the uniqueness of seriniquinone as a promising drug candidate.

Another important investigation for the translation of in vitro results to human use is the prediction of the interaction between drugs and cytochrome P450 (CYP450) enzymes. This pharmacokinetic characterization is necessary once this enzymatic group is responsible for the metabolism of many xenobiotic and endogenous compounds, mainly resulting in their inactivation. This way, we can predict the drug–drug interactions and the influences of the patient’s genetic polymorphism, favoring the design of safer therapeutic regimens [42]. Silva et al. [43] evaluated the interaction of seriniquinone with CYP450 and observed a strong inhibition of CYP1A2, CYP2E1, and CYP3A, as well as a moderate effect on CYP2C19. In addition, seriniquinone was shown to be an irreversible and time-dependent inhibitor of CYP1A2 and CYP3A. Therefore, interactions with other drugs that are metabolized by these enzymes are expected, which may lead to significant toxicity.

## 5. Conclusions

The previous section discussed the pharmacological characteristics and benefits known to date regarding seriniquinone, as well as all of the experiments that were performed in vitro. Advancing these studies requires in vivo tests. However, seriniquinone has low water solubility (0.06 µM) [1], which hampers an investigative progress. Therefore, two paths were taken: Medicinal chemistry (as discussed above) and nanotechnology application, strategies that generated formulations providing improved seriniquinone effects for melanoma treatment. The first strategy was explored by Hammons et al. [1] and Hirata et al. [31], in which chemical modifications on the basic seriniquinone structure were completed to enhance solubility. The new synthetic analogues were more water-soluble, maintained or improved the cytotoxicity and selectivity for melanoma cells, and preserved the molecular target (dermcidin protein), allowing for further investigations with these analogues in vivo.

The use of a nanotechnology-based approach to solubilize seriniquinone emerged based on other reported cases. There are several formulation solutions that nanotechnology can provide. Drugs that are highly toxic and/or poor water-soluble can be formulated into approved nanoformulations. Two classic examples of this are doxorubicin and paclitaxel, both anticancer and lipophilic natural products. Doxorubicin is a DNA intercalating drug, with cardiotoxicity as its main side effect. This was prevented by the use of Doxil^®^ liposomes, the first US FDA approved nanoformulation in 1995 [44]. Paclitaxel, a microtubule-stabilizing drug, could only be dissolved in polyoxyethylated castor oil (Cremophor^®^ EL), resulting in its first formulation labeled Taxol^®^. However, this vehicle is responsible for serious side effects, such as neurotoxicity, hypersensitivity, and nephrotoxicity. In 2005, the US FDA approved the paclitaxel albumin-bound nanoparticle (Abraxane^®^), a Cremophor^®^ EL-free formulation that solved the problem of solubility and provided a safer drug [45]. These examples show that nanotechnology is a promising approach that has the potential to solubilize and deliver seriniquinone, expediting its advance to preclinical studies and future clinical trials, as seen in unpublished data and discussed by Apolinario et al. [46].

**Figure 7:**
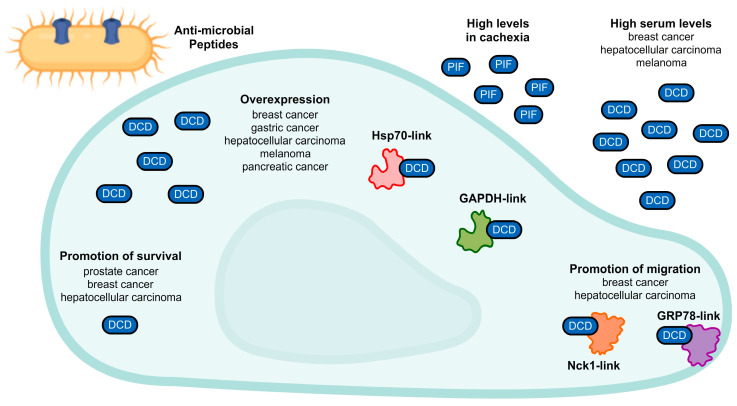
Multiple roles of dermcidin in cancer cells. In addition to the well-described role in skin defense with antimicrobial peptides, dermcidin (DCD) may be involved in a few processes of many types of cancer cells, as illustrated. Proteolysis-inducing factor (PIF), another DCD derivative, is related to cachexia in cancer.

Beyond the anticancer potential of seriniquinone, we have a long way to elucidate the role of its target, dermcidin. This protein is well-established in skin immunity: The full-length protein (110 aa) is cleaved in many antimicrobial peptides that are constitutively secreted by eccrine sweat glands and play an important role in innate immunity by controlling pathogen infections in human skin [47]. Moreover, YP30 is derived from dermcidin, but identified as a neuron survival-promoting peptide [48]. In addition, proteolysis-inducing factor (PIF) is a proteolytic glycoprotein, which is identified as an inducer of cachexia in cancer through severe weight-loss and muscle degradation [49,50]. Outside of this context, more recent data have associated the dermcidin protein with tumorigenesis. Initial evidence reported by Porter et al. revealed that the protein was overexpressed in 10% of invasive breast carcinomas and conferred cell growth and survival, launching dermcidin as a candidate oncogene [51]. Thereafter, several research groups reported additional evidence concerning this protein in various types of cancers, as illustrated in Figure 7: Intracellular or extracellular overexpression of dermcidin in breast cancer [52], gastric cancer [53,54], hepatocellular carcinoma [55,56], lung cancer [57,58], melanoma [59,60], and pancreatic cancer [61]; promotion of cell survival [48,51,62,63,64]; and promotion of cell migration [55,65]. Although there are solid data that link dermcidin and cancer, the proper role of the protein at molecular levels and its contribution to tumorigenesis is still unclear. Therefore, it confounds the prediction and elucidation of a comprehensive mechanism of action for seriniquinone. Nevertheless, it was observed by Trzoss et al. that dermcidin could bind HSP70 and GADPH proteins [2]. Moreover, Shen et al. revealed that dermcidin interacts with NCK1 [55], while Lager et al. showed a link between dermcidin and cell surface GRP78 [65]. However, the mentioned proteins lack a clear correlation with each other and, to date, no studies have explored this type of event. Nevertheless, we may know only a small part of the scenario of seriniquinone, but this complexity only brings it closer to a successful drug candidate.

## References

[1] Hammons J.C., Trzoss L., Jimenez P.C., Hirata A.S., Costa-Lotufo L.V., La Clair J.J., Fenical W. (2019). Advance of Seriniquinone Analogues as Melanoma Agents. ACS Med. Chem. Lett..

[2] Trzoss L., Fukuda T., Costa-Lotufo L.V., Jimenez P., La Clair J.J., Fenical W. (2014). Seriniquinone, a selective anticancer agent, induces cell death by autophagocytosis, targeting the cancer-protective protein dermcidin. Proc. Natl. Acad. Sci. USA.

[3] Ishida K., Tanaka T., Nagai K., Furuichi Y., Terahara T., Anda M., Tsukamasa Y., Fukuda T. (2022). New dihydronaphthothiophene derivatives by the biological transformation of seriniquinone using marine-derived actinomycete Streptomyces albogriseolus OM27-12. J. Antibiot..

[4] Alexander M.D., Burkart M.D., Leonard M.S., Portonovo P., Liang B., Ding X., Joullié M.M., Gulledge B.M., Aggen J.B., Chamberlin A.R. (2006). A Central Strategy for Converting Natural Products into Fluorescent Probes. ChemBioChem.

[5] Hughes C.C., MacMillan J.B., Gaudêncio S.P., Fenical W., La Clair J.J. (2009). Ammosamides A and B Target Myosin. Angew. Chemie Int. Ed..

[6] IARC The Global Cancer Observatory (Globocan) 2020 Database. https://gco.iarc.fr/today/data/factsheets/cancers/16-Melanoma-of-skin-fact-sheet.pdf.

[7] Davis L.E., Shalin S.C., Tackett A.J. (2019). Current state of melanoma diagnosis and treatment. Cancer Biol. Ther..

[8] Lee C., Collichio F., Ollila D., Moschos S. (2013). Historical review of melanoma treatment and outcomes. Clin. Dermatol..

[9] Wagner D.E., Ramirez G., Weiss A.J., Hill G. (1972). Combination Phase 1–II Study of Imidazole Carboxamide (NCS 45388). Oncology.

[10] Hill G.J., Ruess R., Berris R., Philpott G.W., Parkin P. (1974). Chemotherapy of Malignant Melanoma with Dimethyl Triazeno lmidazole Carboxamide (DITC) and Nitrosourea Derivatives (BCN U, CCNU). Ann. Surg..

[11] Schadendorf D., Vaubel J., Livingstone E., Zimmer L. (2012). Advances and perspectives in immunotherapy of melanoma. Ann. Oncol..

[12] Larkin J., Chiarion-Sileni V., Gonzalez R., Grob J.-J., Rutkowski P., Lao C.D., Cowey C.L., Schadendorf D., Wagstaff J., Dummer R. (2019). Five-Year Survival with Combined Nivolumab and Ipilimumab in Advanced Melanoma. N. Engl. J. Med..

[13] Sharma P., Allison J.P. (2015). Immune Checkpoint Targeting in Cancer Therapy: Toward Combination Strategies with Curative Potential. Cell.

[14] Davies H., Bignell G.R., Cox C., Stephens P., Edkins S., Clegg S., Teague J., Woffendin H., Garnett M.J., Bottomley W. (2002). Mutations of the BRAF gene in human cancer. Nature.

[15] Mishra H., Mishra P.K., Ekielski A., Jaggi M., Iqbal Z., Talegaonkar S. (2018). Melanoma treatment: From conventional to nanotechnology. J. Cancer Res. Clin. Oncol..

[16] Curl P., Vujic I., van ‘t Veer L.J., Ortiz-Urda S., Kahn J.G. (2014). Cost-Effectiveness of Treatment Strategies for BRAF-Mutated Metastatic Melanoma. PLoS ONE.

[17] Bhatia S., Tykodi S.S., Thompson J.A. (2009). Treatment of metastatic melanoma: An overview. Oncology (Williston Park).

[18] Oh A., Tran D.M., McDowell L.C., Keyvani D., Barcelon J.A., Merino O., Wilson L. (2017). Cost-Effectiveness of Nivolumab-Ipilimumab Combination Therapy Compared with Monotherapy for First-Line Treatment of Metastatic Melanoma in the United States. J. Manag. Care Spec. Pharm..

[19] Wolchok J.D., Chiarion-Sileni V., Gonzalez R., Rutkowski P., Grob J.-J., Cowey C.L., Lao C.D., Wagstaff J., Schadendorf D., Ferrucci P.F. (2017). Overall Survival with Combined Nivolumab and Ipilimumab in Advanced Melanoma. N. Engl. J. Med..

[20] da Rocha Dias S., Salmonson T., van Zwieten-Boot B., Jonsson B., Marchetti S., Schellens J.H.M., Giuliani R., Pignatti F. (2013). The European Medicines Agency review of vemurafenib (Zelboraf^®^) for the treatment of adult patients with BRAF V600 mutation-positive unresectable or metastatic melanoma: Summary of the scientific assessment of the Committee for Medicinal Products for Huma. Eur. J. Cancer.

[21] Long G.V., Flaherty K.T., Stroyakovskiy D., Gogas H., Levchenko E., de Braud F., Larkin J., Garbe C., Jouary T., Hauschild A. (2017). Dabrafenib plus trametinib versus dabrafenib monotherapy in patients with metastatic BRAF V600E/K-mutant melanoma: Long-term survival and safety analysis of a phase 3 study. Ann. Oncol..

[22] Lugowska I., Kosela-Paterczyk H., Kozak K., Rutkowski P. (2015). Trametinib: A MEK inhibitor for management of metastatic melanoma. Onco. Targets. Ther..

[23] Shih V., Ten Ham R.M., Bui C.T., Tran D.N., Ting J., Wilson L. (2015). Targeted Therapies Compared to Dacarbazine for Treatment of BRAF(V600E) Metastatic Melanoma: A Cost-Effectiveness Analysis. J. Skin Cancer.

[24] Fairlie W.D., Tran S., Lee E.F. (2020). Crosstalk between apoptosis and autophagy signaling pathways. Int. Rev. Cell Mol. Biol..

[25] Huang T., Song X., Yang Y., Wan X., Alvarez A.A., Sastry N., Feng H., Hu B., Cheng S.-Y. (2018). Autophagy and Hallmarks of Cancer. Crit. Rev. Oncog..

[26] Rahmati M., Ebrahim S., Hashemi S., Motamedi M., Moosavi M.A. (2020). New insights on the role of autophagy in the pathogenesis and treatment of melanoma. Mol. Biol. Rep..

[27] Zhang J., Tang P., Zou L., Zhang J., Chen J., Yang C., He G., Liu B., Liu J., Chiang C.M. (2021). Discovery of Novel Dual-Target Inhibitor of Bromodomain-Containing Protein 4/Casein Kinase 2 Inducing Apoptosis and Autophagy-Associated Cell Death for Triple-Negative Breast Cancer Therapy. J. Med. Chem..

[28] Biggers J.W., Nguyen T., Di X., Gupton J.T., Henderson S.C., Emery S.M., Alotaibi M., White K.L., Brown R., Almenara J. (2013). Autophagy, cell death and sustained senescence arrest in B16/F10 melanoma cells and HCT-116 colon carcinoma cells in response to the novel microtubule poison, JG-03-14. Cancer Chemother. Pharmacol..

[29] Liu Y., Wang M., Wang D., Li X., Wang W., Lou H., Yuan H. (2016). Malformin A1 promotes cell death through induction of apoptosis, necrosis and autophagy in prostate cancer cells. Cancer Chemother. Pharmacol..

[30] Mizushima N., Levine B., Cuervo A.M., Klionsky D.J. (2008). Autophagy fights disease through cellular self-digestion. Nature.

[31] Hirata A.S., Rezende-Teixeira P., Machado-Neto J.A., Jimenez P.C., Clair J.J.L., Fenical W., Costa-Lotufo L.V. (2021). Seriniquinones as Therapeutic Leads for Treatment of BRAF and NRAS Mutant Melanomas. Molecules.

[32] Heppt M.V., Siepmann T., Engel J., Schubert-Fritschle G., Eckel R., Mirlach L., Kirchner T., Jung A., Gesierich A., Ruzicka T. (2017). Prognostic significance of BRAF and NRAS mutations in melanoma: A German study from routine care. BMC Cancer.

[33] Ryabaya O.O., Inshakov A.N., Egorova A.V., Emelyanova M.A., Nasedkina T.V., Zasedatelev A.S., Khochenkov D.A., Stepanova E.V. (2017). Autophagy inhibitors chloroquine and LY294002 enhance temozolomide cytotoxicity on cutaneous melanoma cell lines in vitro. Anticancer. Drugs.

[34] Ma X.-H., Piao S.-F., Dey S., Mcafee Q., Karakousis G., Villanueva J., Hart L.S., Levi S., Hu J., Zhang G. (2014). Targeting ER stress–induced autophagy overcomes BRAF inhibitor resistance in melanoma. J. Clin. Invest..

[35] Rangwala R., Leone R., Chang Y.C., Fecher L.A., Schuchter L.M., Kramer A., Tan K.-S., Heitjan D.F., Rodgers G., Gallagher M. (2014). Phase I trial of hydroxychloroquine with dose-intense temozolomide in patients with advanced solid tumors and melanoma. Autophagy.

[36] Strohecker A.M., White E. (2014). Targeting Mitochondrial Metabolism by Inhibiting Autophagy in BRAF-Driven Cancers. Cancer Discov..

[37] Xie X., Koh J.Y., Price S., White E., Mehnert J.M. (2015). Atg7 Overcomes Senescence and Promotes Growth of BrafV600E-Driven Melanoma. Cancer Discov..

[38] Yu Y., Xie Y., Cao L., Yang L., Yang M., Lotze M.T., Zeh H.J., Kang R., Tang D. (2015). The ferroptosis inducer erastin enhances sensitivity of acute myeloid leukemia cells to chemotherapeutic agents. Mol. Cell. Oncol..

[39] Zheng Z.-Y., Li J., Li F., Zhu Y., Cui K., Wong S.T., Chang E.C., Liao Y.-H. (2018). Induction of N-Ras degradation by flunarizine-mediated autophagy. Sci. Rep..

[40] Bolton J.L., Dunlap T. (2017). Formation and Biological Targets of Quinones: Cytotoxic versus Cytoprotective Effects. Chem. Res. Toxicol..

[41] Lown J.W. (1983). The mechanism of action of quinone antibiotics. Mol. Cell. Biochem..

[42] Manikandan P., Nagini S. (2018). Cytochrome P450 Structure, Function and Clinical Significance: A Review. Curr. Drug Targets.

[43] Moreira da Silva R., Carrão D.B., Habenschus M.D., Jimenez P.C., Lopes N.P., Fenical W., Costa-Lotufo L.V., de Oliveira A.R.M. (2020). Prediction of seriniquinone-drug interactions by in vitro inhibition of human cytochrome P450 enzymes. Toxicol. In Vitro.

[44] Barenholz Y. (2012). Doxil^®^—The first FDA-approved nano-drug: Lessons learned. J. Control. Release.

[45] Bernabeu E., Cagel M., Lagomarsino E., Moretton M., Chiappetta D.A. (2017). Paclitaxel: What has been done and the challenges remain ahead. Int. J. Pharm..

[46] Apolinário A.C., Hirata A.S., Anjos Miguel R.D., Costa-Lotufo L.V., Pessoa A., La Clair J.J., Fenical W., Lopes L.B. (2020). Exploring the benefits of nanotechnology for cancer drugs in different stages of the drug development pipeline. Nanomedicine.

[47] Schittek B., Hipfel R., Sauer B., Bauer J., Kalbacher H., Stevanovic S., Schirle M., Schroeder K., Blin N., Meier F. (2001). Dermcidin: A novel human antibiotic peptide secreted by sweat glands. Nat. Immunol..

[48] Cunningham T.J., Hodge L., Speicher D., Reim D., Tyler-Polsz C., Levitt P., Eagleson K., Kennedy S., Wang Y. (1998). Identification of a Survival-Promoting Peptide in Medium Conditioned by Oxidatively Stressed Cell Lines of Nervous System Origin. J. Neurosci..

[49] Cariuk P., Lorite M., Todorov P., Field W., Wigmore S., Tisdale M. (1997). Induction of cachexia in mice by a product isolated from the urine of cachectic cancer patients. Br. J. Cancer.

[50] Lorite M., Thompson M., Drake J., Carling G., Tisdale M. (1998). Mechanism of muscle protein degradation induced by a cancer cachectic factor. Br. J. Cancer.

[51] Porter D., Weremowicz S., Chin K., Seth P., Keshaviah A., Lahti-Domenici J., Bae Y.K., Monitto C.L., Merlos-Suarez A., Chan J. (2003). A neural survival factor is a candidate oncogene in breast cancer. Proc. Natl. Acad. Sci. USA.

[52] Brauer H.A., D’Arcy M., Libby T.E., Thompson H.J., Yasui Y.Y., Hamajima N., Li C.I., Troester M.A., Lampe P.D. (2014). Dermcidin expression is associated with disease progression and survival among breast cancer patients. Breast Cancer Res. Treat..

[53] Deans D.A.C., Wigmore S.J., Gilmour H., Tisdale M.J., Fearon K.C.H., Ross J.A. (2006). Expression of the proteolysis-inducing factor core peptide mRNA is upregulated in both tumour and adjacent normal tissue in gastro-oesophageal malignancy. Br. J. Cancer.

[54] Zhang J., Ding W., Kuai X., Ji Y., Zhu Z., Mao Z., Wang Z. (2018). Dermcidin as a novel binding protein of lncRNA STCAT3 and its effect on prognosis in gastric cancer. Oncol. Rep..

[55] Shen S.-L., Qiu F.-H., Dayarathna T.K., Wu J., Kuang M., Li S.S.C., Peng B.-G., Nie J. (2011). Identification of Dermcidin as a novel binding protein of Nck1 and characterization of its role in promoting cell migration. Biochim. Biophys. Acta.

[56] Qiu F., Qiu F., Liu L., Liu J., Xu J., Huang X. (2018). The Role of Dermcidin in the Diagnosis and Staging of Hepatocellular Carcinoma. Genet. Test. Mol. Biomark..

[57] López-Sánchez L.M., Jurado-Gámez B., Feu-Collado N., Valverde A., Cañas A., Fernández-Rueda J.L., Aranda E., Rodríguez-Ariza A. (2017). Exhaled breath condensate biomarkers for the early diagnosis of lung cancer using proteomics. Am. J. Physiol. Cell. Mol. Physiol..

[58] Núñez-Naveira L., Mariñas-Pardo L.A., Montero-Martínez C. (2019). Mass Spectrometry Analysis of the Exhaled Breath Condensate and Proposal of Dermcidin and S100A9 as Possible Markers for Lung Cancer Prognosis. Lung.

[59] Ortega-Martínez I., Gardeazabal J., Erramuzpe A., Sanchez-Diez A., Cortés J., García-Vázquez M.D., Pérez-Yarza G., Izu R., Luís Díaz-Ramón J., de la Fuente I.M. (2016). Vitronectin and dermcidin serum levels predict the metastatic progression of AJCC I–II early-stage melanoma. Int. J. Cancer.

[60] Mancuso F., Lage S., Rasero J., Díaz-Ramón J.L., Apraiz A., Pérez-Yarza G., Ezkurra P.A., Penas C., Sánchez-Diez A., García-Vazquez M.D. (2020). Serum markers improve current prediction of metastasis development in early-stage melanoma patients: A machine learning-based study. Mol. Oncol..

[61] Stewart G.D., Skipworth R.J.E., Pennington C.J., Lowrie A.G., Deans D.A.C., Edwards D.R., Habib F.K., Riddick A.C.P., Fearon K.C.H., Ross J.A. (2008). Variation in dermcidin expression in a range of primary human tumours and in hypoxic/oxidatively stressed human cell lines. Br. J. Cancer.

[62] Stewart G.D., Lowrie A.G., Riddick A.C.P., Fearon K.C.H., Habib F.K., Ross J.A. (2007). Dermcidin expression confers a survival advantage in prostate cancer cells subjected to oxidative stress or hypoxia. Prostate.

[63] Bancovik J., Moreira D.F., Carrasco D., Yao J., Porter D., Moura R., Camargo A., Fontes-Oliveira C.C., Malpartida M.G., Carambula S. (2015). Dermcidin exerts its oncogenic effects in breast cancer via modulation of ERBB signaling. BMC Cancer.

[64] Lowrie A.G., Wigmore S.J., Wright D.J., Waddell I.D., Ross J.A. (2006). Dermcidin expression in hepatic cells improves survival without N-glycosylation, but requires asparagine residues. Br. J. Cancer.

[65] Lager T.W., Conner C., Keating C.R., Warshaw J.N., Panopoulos A.D. (2021). Cell surface GRP78 and Dermcidin cooperate to regulate breast cancer cell migration through Wnt signaling. Oncogene.

